# RNA modifications in cancer immune therapy: regulators of immune cells and immune checkpoints

**DOI:** 10.3389/fimmu.2024.1463847

**Published:** 2024-09-20

**Authors:** Xiangyu Qin, Huali Liu, Qixuan Zhang, Yuhang Che, Tianyu Lei, Fang Tang, Qinyong Hu

**Affiliations:** ^1^ Department of Oncology, Renmin Hospital of Wuhan University, Wuhan, China; ^2^ Renmin Hospital of Wuhan Economic and Technological Development Zone (Hannan), Wuhan, China; ^3^ Wuhan University Heavy Ion Medicine Center, Wuhan, China; ^4^ Reproductive Medicine Center, Renmin Hospital of Wuhan University, Wuhan, China; ^5^ Department of Radiation and Medical Oncology, Zhongnan Hospital of Wuhan University, Wuhan, China

**Keywords:** RNA modification, cancer, immune checkpoint, immune cell, immune therapy

## Abstract

RNA modifications are epigenetic changes that alter the structure and function of RNA molecules, playing a crucial role in the onset, progression, and treatment of cancer. Immune checkpoint inhibitor (ICI) therapies, particularly PD-1 blockade and anti-CTLA-4 treatments, have changed the treatment landscape of virous cancers, showing great potential in the treatment of different cancer patients, but sensitivity to these therapies is limited to certain individuals. This review offers a comprehensive survey of the functions and therapeutic implications of the four principal RNA modifications, particularly highlighting the significance of m6A in the realms of immune cells in tumor and immunotherapy. This review starts by providing a foundational summary of the roles RNA modifications assume within the immune cell community, focusing on T cells, NK cells, macrophages, and dendritic cells. We then discuss how RNA modifications influence the intricate regulatory mechanisms governing immune checkpoint expression, modulation of ICI efficacy, and prediction of ICI treatment outcomes, and review drug therapies targeting genes regulated by RNA modifications. Finally, we explore the role of RNA modifications in gene editing, cancer vaccines, and adoptive T cell therapies, offering valuable insights into the use of RNA modifications in cancer immunotherapy.

## Introduction

RNA modification is an epigenetic change that alters the structure and function of RNA molecules by inserting, deleting, or substituting nucleotides at specific locations, playing a key role in cellular physiology and pathology. Currently, the roster of RNA modifications that have been pinpointed has extended to over 170 distinct subtypes (1), including methylation, acetylation, pseudouridinization, among others.

RNA methylation modifications are regulated by specific proteins, including methyltransferase “writers” that add marks, demethylase “erasers” that remove them, and “readers” that recognize these modifications. Among RNA modifications, N1-methyladenosine (m1A) (2), 5-methylcytosine (m5C) (3), N6-methyladenosine (m6A) (4), and 7-methylguanosine (m7G) have been the focus of comprehensive studies regarding their oncogenic properties and are viewed as key determinants in the occurrence (5), development, and treatment of tumors.

Immune cells within tumors play a key role in clearing the tumor. Activating immune cells, especially the tumor-clearing functions of T cells, can suppress tumor growth. However, immune cells in the tumor microenvironment are typically in a state of suppression, leading to immune evasion. We have found that RNA modifications are involved in the regulation of immune cell suppression in tumors. Immunotherapy, such as anti-PD-1/PD-L1 therapy, has been widely applied in clinical practice, but only a subset of patients is sensitive to immunotherapy. The efficacy of cancer immunotherapy is contingent upon a multitude of factors, including the type of tumor, tumor’s mutational burden, the stability of microsatellites, and the combined use of chemotherapy drugs. Notably, the presence of immune cell infiltration within the tumor microenvironment and the expression of immune checkpoints on the tumor’s surface play pivotal roles in the therapy’s success. We have found that RNA modifications, primarily the m6A modification, have a complex regulatory effect on the tumor infiltration of immune cells and the expression of PD-L1. By targeting the m6A regulators in tumors or immune cells, it is possible to increase immune cell infiltration, regulate the expression of PD-L1, sensitize patients to immunotherapy, inhibit tumor growth, and ultimately improve patient prognosis.

We have compiled a summary of the regulatory roles of four types of RNA methylation modifications, mainly m6A modification, in immune cells, as well as their regulatory effects on the expression of PD-L1 in tumor cells and the research progress on m6A regulators as therapeutic targets. We also reported on the predictive role of methylation modification-related genes in the efficacy of immunotherapy.

## Overview of RNA modifications

**Figure 1:**
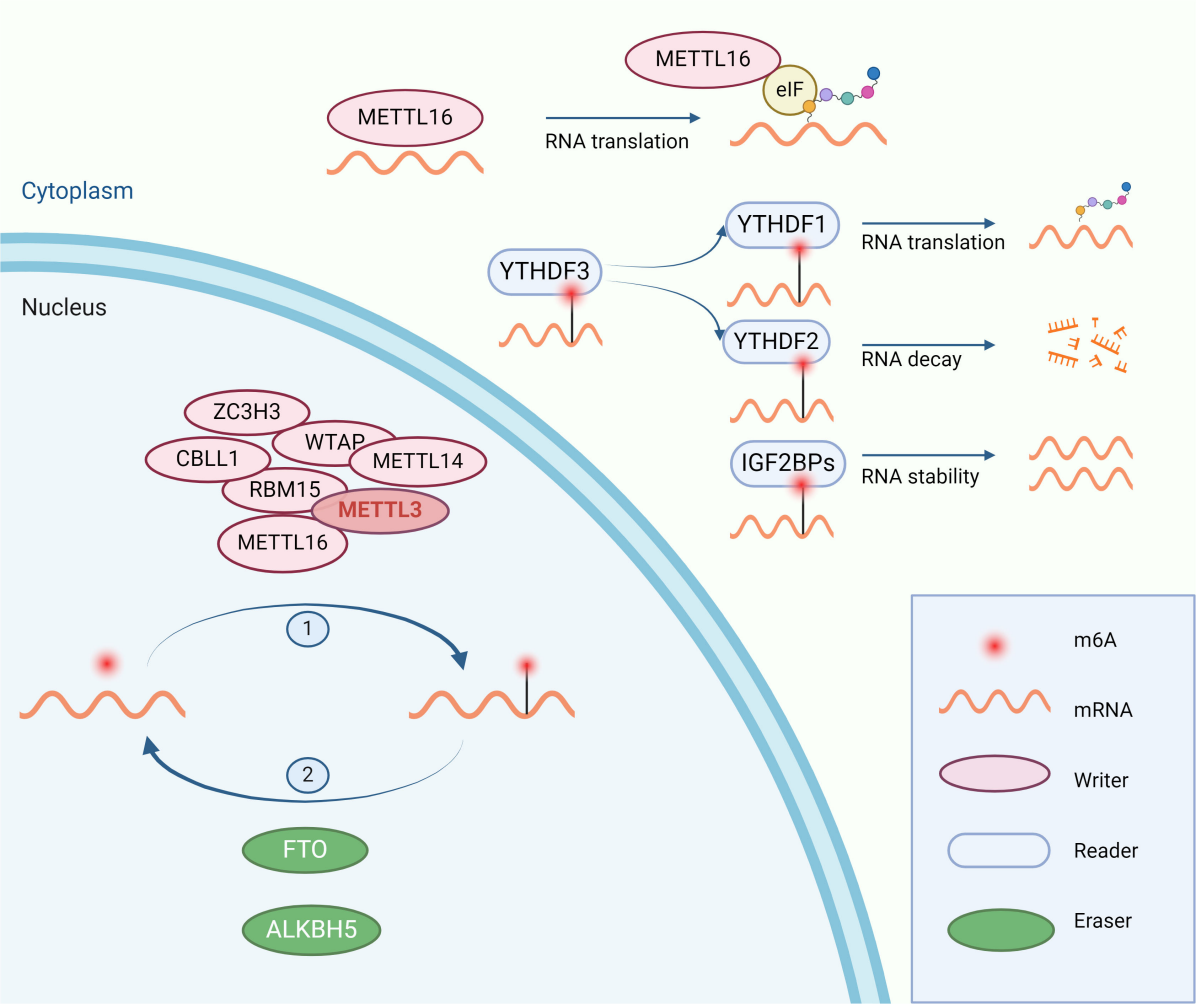
The process of m6A modification: m6A modification is catalyzed by “writers” in the nucleus and regulates the stability, translation, and degradation of RNA after being recognized by “readers” in the cytoplasm. The m6A modification is removed by “erasers”.

m6A, representing N6-adenosine methylation, is recognized as the most frequent and plentiful type of post-transcriptional RNA modification, predominantly occurring within mRNA in the nucleus (6). This modification contributes to essential life processes such as hematopoiesis, central nervous system development, and germ cell differentiation (4). Common regulatory enzymes include “writers” METTL3, METTL14, METTL16, “readers” YTHDF1, YTHDF2, YTHDF3, LRPPRC, IGF2BP1 and IGF2BP3, among others, and “erasers” FTO, ALKBH5. METTL3 is the most critical ‘writer’ enzyme for m6A modification, serving as the catalytic subunit of the methyltransferase complex that mediates N6-methyladenosine (m6A) methylation on mRNA. It commonly forms a heterodimer with METTL14, facilitating the process (7). Wilms Tumor 1 Associated Protein (Wtap), a regulatory subunit of the complex, enhances the binding affinity of METTL3 for mRNA (8). METTL3 and METTL14 are primarily located in the nucleus, while METTL16 is mainly found in the cytoplasm. Although METTL16 also mediates the m6A modification, its role in promoting tumorigenesis in various cancers is more noteworthy, which is related to the acceleration of mRNA translation by METTL16 binding to eIF3a/b in the cytoplasm (9). YTHDFs recognize mRNAs modified with m6A; YTHDF1 promotes the translation of these mRNAs, while YTHDF2 accelerates their degradation (10). YTHDF3 interacts with YTHDF1 to augment translation of m6A-modified mRNA and influences the RNA binding activity of YTHDF2 (11). And IGF2BPs enhance the stability of mRNA after recognizing m6A modification (12). FTO and ALKBH5 are the only two demethylases identified for m6A demethylation, which remove the m6A modification from RNA (**Figure 1**). The role of m6A regulatory enzymes in cancer has been widely reported. Several preclinical experiments have demonstrated that the use of drugs targeting m6A regulators can inhibit tumor growth or enhance the therapeutic efficacy of immunotherapy (**Table 1**).

**Table 1:** Drugs targeting m6A and their mechanisms.

Drugs	Targeting Regulators	Cancer type	Function	References
STM2457	METTL3	myeloid leukemia	decrease acute myeloid leukemia growth, increase its differentiation and apoptosis	(13)
miR-4429		gastric cancer	inhibit GC cells proliferation and induce apoptosis	(14)
Metformin		breast cancer	inhibit the miR-483-3p/METTL3/m6A/p21 pathway to suppress proliferation of breast cancer cells	(15)
BTYNB	IGF2BP1	melanoma and ovarian cancer	inhibit the binding of IMP1 to c-Myc mRNA suppresses the proliferation of ovarian cancer and melanoma cells	(16)
Triptonide		nasopharyngeal carcinoma	disrupt Lnc-THOR-IGF2BP1 signaling and inhibit tumor growth	(17)
Ucurbitacin B		HCC	induce tumor cell apoptosis and increase immune cell infiltration by allosterically blocking the interaction between IGF2BP1 and m6A through the KH1–2 domain	(18)
CWI1-2	IGF2BP2	acute myeloid leukemia(AML)	inhibited glutamine absorption and impair mitochondrial function in AML cells, suppress AML progression	(19)
ABCF1 mRNA	IGF2BP3	Ewing’s sarcoma	bind and inhibit IGF2BP3, suppress the growth of Ewing’s sarcoma cells	(20)
Berberine		colorectal cancer	downregulate IGF2BP3 and inhibit PI3K/AKT pathway to inhibit the proliferation of colorectal cancer cells	(21)
LNP-iYthdf1	YTHDF1	nonalcoholic steatohepatitis-related hepatocellular carcinoma	enhance the efficacy of PD-1 blockade therapy	(22)
DF-A7	YTHDF2	colon adenocarcinoma and melanoma	promote the infiltration and M2 polarization of CX3CR1^+^ macrophages, inhibit glycolysis in tumor cells, and enhance the effector functions of CD8^+^ T cells	(23)
JX5		T-cell acute lymphoblastic leukemia	bind to the IGF2BP2 KH3-4 domain and inhibit proliferation of tumor cells	(24)
CS1 and CS2	FTO	AML	inhibit FTO activity and signaling thereby suppresses the viability of AML cells	(25)
Saikosaponin D			inhibit proliferation of AML cells, promote cells apoptosis and cycle arrest	(26)
R-2HG			target the FTO/m6A/MYC/CEBPA signaling to inhibit proliferation of AML cells	(27)
FB23-2			inhibit proliferation of AML cells	(28)
Dac51		melanoma	increase infiltration and cytotoxicity of CD4^+^T cells, inhibit tumor growth, and have a synergistic effect with anti-PD-L1 blockade	(29)
MV1035	ALKBH5	Glioblastoma	inhibit ALKBH5, thereby suppress the migration and invasiveness of tumor cells	(30)
ALK-04		melanoma	inhibit Mct4/Slc16a3 expression, lactate content, Treg cells MDSC in TME and enhance the efficacy of GVAX/anti-PD-1 therapy	(31)
Hiram	TRMT6/TRMT61A	HCC	inhibit the growth of HCC	(32)

m1A and m5C modifications predominantly occur in mRNA, tRNA, and rRNA. The m1A modification is mainly found in tRNA and shares some similarities with m6A modification in mRNA, although it occurs at a much lower frequency. The similarities between m1A and m6A modifications are also reflected in their shared binding proteins YTHDF1, YTHDF2, YTHDF3, and the demethylase FTO (2). m5C plays an important role in maintaining the structure and stability of tRNA and rRNA. m5C modification can occur in mRNA and can regulate the stability of mRNA, but the research on m5C in mRNA is not sufficiently in-depth and comprehensive (3). m7G is widely present in mRNA and is crucial in the translation process and is involved in many important physiological processes (5).

The emergence of new sequencing technologies and bioinformatics tools has facilitated the detection and in-depth understanding of RNA modifications. However, there are still many RNA modifications that have not yet been discovered, and research on many that have been confirmed is far from adequate. We have primarily focused on the function of m6A modification, as it is the most prevalent and well-studied modification in mRNA, but it is important to recognize that other RNA modifications also hold significant potential. The relationship between RNA modifications and the occurrence and development of tumors, as well as their therapy, is currently a hotbed of research. Multiple regulators of RNA modifications are considered oncogenes and have shown potential therapeutic value. The exploration of gene editing technology to enhance RNA modifications that combat tumors and reduce those that promote tumor growth also presents a valuable research avenue.

## RNA modifications in immune cells

### T cells

**Figure 2:**
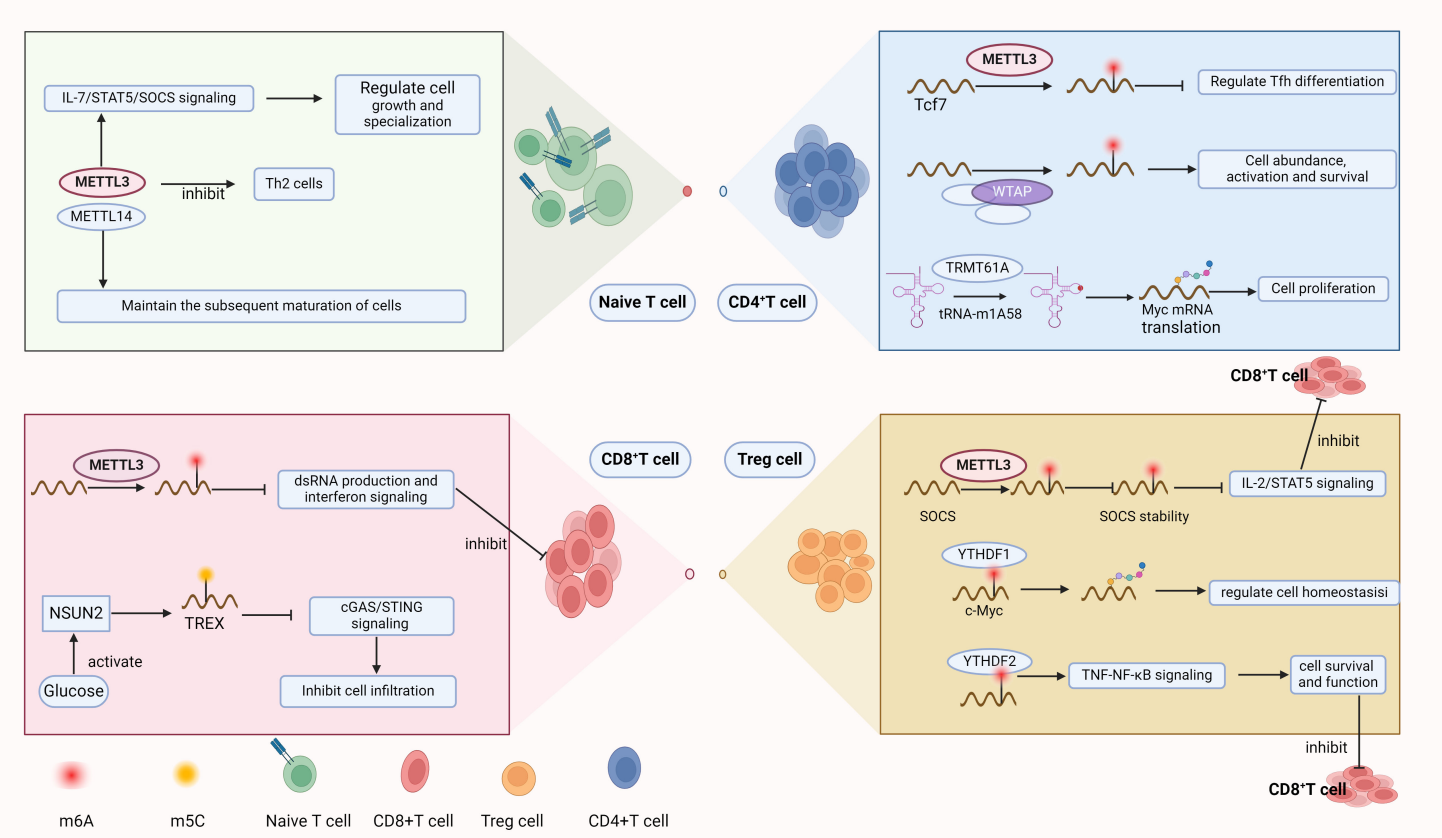
RNA modification regulates the development of Naive T cells and can exert regulatory effects in various T cell subsets.

T cells have been pivotal in the ongoing oversight and extermination of tumors (33), and their function within tumors is regulated by RNA modifications (**Figure 2**). The m6A methylation catalyzed by METTL3 is essential for preserving T cell stability and directing their differentiation, with influence over the growth and specialization of naive T cells through the IL-7/STAT5/SOCS axis. Removal of METTL3 from T cells leads to a contraction of the Th17 and Th1 lineages and an escalation in the Th2 cell presence among naive T cells, contrasting with the METTL14 knockout, which impedes T cell maturation beyond the naive stage (34). The m6A/ALKBH5 mechanism is instrumental in controlling the equilibrium of γδ T cell development. ALKBH5 modulates the signaling of Jagged1/Notch2 by removing m6A modifications on them, thereby imposing a restraint on the developmental progression and the lineage specification of γδ T cells (35). Moreover, METTL3 dictates the progression and role of iNKT cells through the METTL3/m6A/Creb1 axis. Deletion of METTL3 impairs the proliferation, differentiation, and cytokine secretion of iNKT cells, leading to a deficiency in tumor resistance (36).

In CD4^+^ T cells, the m1A modification (tRNA-m1A58) at the 58th nucleotide position within the tRNA sequence, mediated by the “writer” protein TRMT61A, ensures the efficient translation of Myc mRNA, thereby supporting the propagation and the functional diversification of CD4^+^ T cells (37). The expression levels of m5C “writer” NSUN3 and NSUN4 are positively correlated with CD8^+^ T cell infiltration, and NSUN4 expression levels are positively associated with the presence of CD4^+^ T cells (38). The expression level of NSUN2 is also related to CD4^+^ T cells and may be involved in the regulation of CD4 memory T cells (39). Regulatory T cells (Treg), a specialized subset of CD4^+^ T lymphocytes, are pivotal in sustaining immune tolerance and averting autoimmune responses. In tumors, Treg cells can suppress immune responses within the tumor, leading to immune evasion (40). Mettl3 contributes to the upregulation of the IL-2/STAT5 pathway via its capacity to catalyze m6A modifications, regulating members of the SOCS family, thereby maintaining the function and stability of Treg cells and promoting T cell suppression (41). YTHDF1 reads the m6A-modified c-Myc mRNA, thereby regulating the translation and expression of c-Myc in Treg cells, and thus coordinating Treg homeostasis (42). YTHDF2 is involved in the regulation of Treg cells in the tumor microenvironment (TME), maintaining the survival and function of Treg cells by controlling the TNF-NF-κB signaling pathway within Treg cells. The absence of YTHDF2 in Treg cells of the tumor microenvironment leads to an increase in CD8^+^ T cell infiltration and the expansion of the antitumor CD4^+^ TH1 subset (43). In squamous cell carcinoma of the head and neck, METTL1 deletion leads to a substantial reduction in Treg cells and the amelioration of CD4^+^ T cell exhaustion. This indicates that METTL1-mediated m7G modulates immune infiltration by regulating the levels of Treg cells (44, 45). Follicular helper T (Tfh) cells, a specialized subset of CD4^+^ T cells, are instrumental in the orchestration of humoral immune responses. METTL3 regulates the Tcf7 transcript through m6A modification, ensuring proper differentiation and development of TFH (46). As an indispensable element of the heterodimeric methyltransferase complex, Wtap also participats in the regulation of T cells. The knockout of WTAP leads to a reduction in the abundance and spontaneous activation of peripheral CD4^+^ and CD8^+^ T cells and eliminates the expansion effect induced by the T cell receptor (TCR). Additionally, Wtap is essential for the regulatory control of apoptosis in CD4^+^ T cells, impacting their survival upon TCR engagement (47).

The absence of YTHDF2 in tumors impairs tumor glycolytic metabolism, therefore enhancing the mitochondrial respiration of CD8^+^ T cells to strengthen antitumor capabilities (23). A pan-cancer examination has shown that METTL1, an enzyme responsible for m7G methylation, is positively correlated with Treg cell numbers in diverse cancer subtypes. The m7G methyltransferase WBSCR22 has been shown to regulate the Zac1/p53 pathway to exert a pro-tumorigenic effect and is highly expressed in activated CD8^+^ T cells, indicating that WBSCR22 may participate in the regulation of CD8^+^ T cells (48, 49). The m5C methylation reader YBX1 is positively correlated with CD4^+^ memory T cells, CD8^+^ T cells, type 1 and type 2 T helper cells in pancreatic ductal adenocarcinoma (50). The m5C methyltransferase NSUN2 mediates m5C modification of TREX2 transcripts after being activated by glucose, inhibiting the activation of cGAS/STING, thus inhibiting the infiltration of CD8^+^ T cells (51). Inhibition of METTL3 leads to an increase in dsRNA formation, which in turn enhances interferon signaling and augments the capacity of T cells to eliminate cancer cells (52). Similarly, the knockout of Mettl14 leads to a significant reduction in iNKT cells mediated by the p53 apoptosis pathway (53). Additionally, m6A modification mediated by Mettl3 in T cells regulates the migration of T cells in an acidic tumor microenvironment. By inducing Mettl3-mediated m6A modification in T cells, the expression of integrin α subunit ITGB1 can be upregulated, thereby enhancing T cell tumor infiltration and antitumor activity, and relieving the suppression of T cells by the acidic microenvironment (54).

### Natural killer cells

As part of the innate immune response, NK cells are essential for monitoring and eliminating cancerous cells, and they represent a key target for cancer immunotherapy (55). The m6A modification plays an indispensable role in maintaining the tumor infiltration and cytotoxicity of NK cells, primarily regulated by METTL3, METTL14, and YTHDF2. METTL3-mediated m6A methylation promotes the maturation, expansion, and functionality of NK cells through the modulation of IL-15 signaling within the AKT-mTOR/MAPK-ERK pathway. Similarly, YTHDF2 in NK cells regulates the proliferation or survival of NK cells after reading the m6A modification of Tardbp, and modulates the expression of cytotoxicity-related molecules through the STAT5-YTHDF2 positive feedback axis, which participates in the survival, proliferation, and terminal maturation of IL-15-mediated NK cells (56). When METTL3 is knocked out in NK cells, the tumor infiltration and the ability to secrete immune factors such as GzmB and INF-γ are significantly decreased, cytotoxicity is markedly reduced, and an increase in expression levels of the inhibitory receptor TIGIT is observed in the TME (57). m6A contributes to mRNA stability and promotes the early activation and effector functions of NK cells by directly modifying important mRNAs such as *Prf1* and *Gzmb*. The mTORC1 supports m6A methylation in NK cells through the c-MYC-MAT2A axis to promote SAM synthesis (58). The expression level of the m5C methyltransferase NSUN2 is associated with the level of resting NK cells and may takes part in the regulation of NK cells (39). The m5C methylation reader YBX1 is found to be positively related to the presence of NK cells in cases of pancreatic ductal adenocarcinoma (50).

### Macrophages

Macrophages are innate immune cells that play a crucial and complex role in tumor immunity, with the antitumor M1 polarization and the pro-tumor M2 polarization being two common differentiations of macrophages in tumor immunity. Tumor-infiltrating macrophages (TAMs) typically acquire the immunosuppressive M2 polarization (59, 60). RNA modifications participate in the regulation of macrophages in various aspects. Tumor-associated macrophages constitute a major part of the cellular composition within the TME. METTL3 promotes the degradation of Irakm mRNA by adding m6A modification, enhancing TLR4 signaling, activating macrophages, and inducing M1 polarization in TAMs, thereby increasing their tumor-killing ability (61). The METTL3 in macrophages increases the stability of STAT1 mRNA transcripts by adding m6A modifications, promoting the M1 polarization of macrophages (62). In myeloid cells, METTL3 activates the NF-κB pathway and STAT3 signaling, leading to M1 and M2-like polarization of macrophages, and fosters the proliferation and spread of cancer cells, contingent upon the infiltration of M1 and M2 phenotype-like TAMs (63), and maintains YTHDF1-mediated SPRED2. Additionally, the FTO demethylase influences the NF-κB pathway, stabilizing STAT1 and PPAR-γ mRNAs, crucial for activating M1 and M2 macrophages. YTHDF2 is involved in this process and antagonizes FTO in regulating the stability of PPAR-γ mRNA (64). IL-4 stimulates an increase in IGF2BP2, which then binds to the m6A-modified TSC1. This binding modulates the signaling through the TSC1/mTORC1 and TSC1/2/MEK/ERK axes, orchestrating the balance between M2 and M1 macrophage states, and driving a transition toward the M2 phenotype within macrophage populations (65). In the tumor lactoacidotic environment, H3K178ac induces the acetylation and upregulation of METTL3 expression. METTL3 mediates m6A modification of the Jack1 mRNA transcript in TIM, which is then read by YTHDF1 to enhance the translation efficiency of JAK1 and the phosphorylation of STAT3, thereby enhancing the immunosuppressive function of TIM (66). The high-risk score of m5C-lncRNA is associated with the high expression of M0 and M2 phenotype macrophages in pancreatic cancer, suggesting that m5C-related lncRNAs may regulate the polarization of macrophages in pancreatic cancer (67). The m7G methyltransferase METTL1 is negatively correlated with M2 and M0 macrophages in tenosynovial giant cell tumors, suggesting that METTL1 may induce M1 polarization of macrophages in tenosynovial giant cell tumors. However, in prostate cancer, METTL1-mediated m7G modification induces M2 polarization of macrophages, indicating the heterogeneity of METTL1 functions in different tumors (44, 68). Additionally, tumor-associated macrophages regulate the expression of CD8^+^ T cells through m6A-associated mechanisms. C1q^+^TAMs specifically express METTL14 and YTHDF2, and maintain the level of tumor infiltration and cytotoxicity of CD8^+^ T cells in a METTL14-dependent manner (69).

### Dendritic cells

Dendritic cells, pivotal in the immune response, function as essential antigen-presenting entities that engage in the acquisition, modification, and conveyance of tumor antigens, as well as in the stimulation of T cell responses (70). In tumor immunity, RNA modifications regulate the antigen cross-presentation of dendritic cells and their subsequent function in activating T cells, as well as the migration of dendritic cells. YTHDF1 boosts the synthesis of lysosomal proteases by recognizing the m6A mark on their mRNA, potentially accelerating the breakdown of tumor antigens internalized by dendritic cells. This action may consequently suppress the dendritic cells’ capacity to initiate a cross-priming response in T cells (61, 71). In studies on gastric cancer, YTHDF1 was shown to not only suppress the recruitment of mature DC cells and T cell activation but also inhibit the expression of MHC II and IL-12 (72). METTL3 facilitates the development and maturation of dendritic cells, as well as the subsequent activation of T cells, through its role in m6A methylation. It boosts the translation efficiency of mRNAs encoding CD40, CD80, and the TLR4-associated signaling molecule Tirap. Additionally, METTL3 amplifies the activity of the TLR4/NF-κB signaling cascade and stimulates the synthesis of cytokines that drive an inflammatory response (73). m6A modification is also involved in the migration of dendritic cells; after being read by YTHDF2, it reduces the expression level of lnc-Dpf3, whose expression negatively regulates the induction of CC-chemokine receptor 7 (CCR7) and the migration of dendritic cells to the draining lymph nodes. Moreover, lnc-Dpf3 forms a complex with HIF-1α, impeding the expression of glycolysis-driven genes under HIF-1α’s control, like Ldha. This interaction curtails the glycolytic activity and the movement potential of DCs (74). The m5C methylation reader YBX1 is related to activated dendritic cells in pancreatic ductal adenocarcinoma (50).

Research on the specific regulators of m1A/m5C/m7G and their relationship with immune cells is limited, but growing evidence suggests that these RNA modifications are involved in the regulation of immune cells in various types of cancer. For instance, in clear cell renal cell carcinoma(ccRCC), the score of m7G-related genes is positively correlated with CD4^+^, CD8^+^ T cells, and Treg cells, M0 macrophages, and negatively correlated with dendritic cells, M2 macrophages, and other immune cells (75). In diffuse large B-cell lymphoma, m5C-related genes regulate the infiltration of eosinophils, Treg cells, and M2 macrophages, and control the activation of T cells by modulating immune checkpoints such as PD-L1 and CTLA-4 (76). In prostate cancer, there are significant expression differences in CD8^+^ T cells, M1 macrophages, and M2 macrophages among the two m5C immune subtypes. In colon cancer, a low m1A score is related to the proliferation of CD8^+^ T effector cells (77). In lung adenocarcinoma, the m1A score is related to all immune cells (78). We have summarized the effects of m6A modification on immune cells that have not been previously mentioned (**Table 2**).

**Table 2:** Regulation of immune cells by m6A modification.

	Regulators	Cancer type	Function	References
Writers	METTL3	colorectal carcinoma	reduce infiltration of CD8^+^ T cells	(79)
			increase recruitment of M2 macrophages	(80)
		colorectal cancer	inhibit CD4^+^ T cells and CD8^+^ T cells by promoting the accumulation of MDSCs	(81)
			facilitate M2 macrophage polarization	(82)
		HCC	reduce infiltration of GZMB^+^ IFN-γ ^+^CD8^+^ T cells	(83)
			promote macrophages recruitment and M2 polarization	(84)
		melanoma	reduce infiltration of CD8^+^ T cells	(79)
		NSCLC	reduce infiltration of CD8^+^ T cells	(85)
		thyroid cancer	reduce M2 macrophages and infiltration of Tregs	(86)
	METTL14	colorectal carcinoma	reduce infiltration of CD8^+^ T cells	(79)
		melanoma	reduce infiltration of CD8^+^ T cells	(79)
		colorectal cancer	maintain the function of CD8^+^ T cells	(69)
		cervical cancer	increase the proportion of PD-1+ TAMs, inhibit the phagocytic function of macrophages	(87)
		lung cancer	inhibit activation and infiltration of CD8^+^ T cells	(88)
	METTL16	HCC	promote macrophages recruitment and M2 polarization	(84)
		pancreatic ductal aenocarcinoma	increase infiltration of CD8^+^ T cells and B cells	(89)
Readers	YTHDF1	HCC	inhibit CD8^+^ T cells by promoting the accumulation of MDSCs	(22)
		prostate cancer	inhibit the cytotoxicity of CD8^+^ T cells	(90)
		colorectal cancer	inhibit CD8^+^ T cells by promoting the migration of MDSCs	(91)
	YTHDF2	bladder cancer	inhibit recruitment of CD8^+^ T cells	(92)
		melanoma	reduce infiltration of CD8^+^ T cells and reduce CD4^+^ Th1 subset	(43)
		colon carcinoma		
		triple-negative breast cancer	promote the pro-tumoral phenotype polarization of macrophages	(93)
	YTHDF3	ccRCC	increase infiltration of CD8^+^ T cells	(94)
		melanoma	inhibit recruitment and antitumoral polarization of macrophages and activation and cytotoxicity of CD8^+^ T cells	(23)
		colon adenocarcinoma		
		thyroid cancer	increase infiltration of CD4^+^ T cells and macrophages	(95)
	IGF2BP1	colon cancer	inhibit the cytotoxicity of CD8^+^ T cells	(96)
		HCC	increase infiltration of CD4^+^, CD8^+^T cells, CD56^+^NK cells and F4/80+ macrophages	(18)
	IGF2BP3	HCC	promote infiltration and M2 polarization of macrophages, inhibit activation of CD8^+^T cells	(97)
	LRPPRC	HCC	reduce infiltration of CD4^+^ and CD8^+^ T cells as well as CD56^+^ NK cells and F4/80^+^ macrophages	(18, 98)
Erasers	FTO	melanoma	restrict activation and function of CD8^+^ T cells	(29)
		HCC	inhibit recruitment and activation of CD8^+^ T cells	(99)
	ALKBH5	colorectal cancer	inhibit CD8^+^ T cells and NK cells by promoting the accumulation of MDSCs	(100)
			promote M2 macrophage polarization	(101)
		intrahepatic cholangiocarcinoma	inhibit the expansion and cytotoxicity of CD8^+^ T cells	(102)
		HCC	increase recruitment of PD-L1^+^ macrophages	(103)
		gioblastoma mltiforme	increase recruitment of tumor-associated macrophages, reduce infiltration of CD3^+^, CD4^+^, CD8^+^ T cells	(104, 105)
		ovarian cancer	promote M2 polarization of macrophages	(106)
		NSCLC	recruit PD-L1^+^ TAMs, promote M2 macrophage polarization	(107)
		lung adenocarcinoma	regulate the polarization of M1/M2 macrophages	(108)

We have reviewed the important role of RNA modifications, especially m6A modification, in regulating immune cells. m6A modification has a profound impact on tumor immune surveillance and the response to immunotherapy by affecting the maturation, differentiation, and function of T cells, NK cells, macrophages, and dendritic cells. These findings not only reveal new regulatory mechanisms of immune cells in the tumor microenvironment but also provide potential targets for the development of new cancer treatment strategies.

Despite significant progress in existing research, there are still some limitations. For example, most studies focus on specific types of cancer, and the universality of RNA modifications across different cancer types and their roles at various stages of tumor development are not yet fully understood. Future research needs to more comprehensively and deeply explore the regulatory effects of RNA modifications on immune cells in different cancer types and assess their potential as biomarkers and therapeutic targets.

## RNA modifications and immune checkpoint

RNA modifications have been shown to be associated with the expression of various immune checkpoints, such as the upregulation of PDCD1 and KIR3DL1 and the downregulation of TIGIT, IDO1, and BTLA in the high-risk group established based on m6A scoring in diffuse large B-cell lymphoma (109). In breast cancer, the groups differentiated by m1A scoring exhibit differential expression of immune checkpoints, for example, TIGIT, IDO1, LAG3, and ICOS (110). We mainly summarize and introduce the research findings related to RNA modifications and PD-1/PD-L1 and CTLA-4.

### PD-L1

PD-L1 is mainly expressed on the surface of tumor cells and suppresses the functions of cytotoxic T cells by binding to PD-1 on the surface of T cells. PD-1/PD-L1 blockade therapy has improved the prognosis of many cancers, yet only a subset of patients are sensitive to anti-PD-1 or PD-L1 treatment, and resistance may occur (111). Across a range of malignancies, the metrics derived from the analysis of m6A, m1A, m5C, and m7G regulatory factors exhibit a substantial association with the levels of PD-L1 protein expression (50, 75, 77, 78, 112–121). Firstly, regulators of m6A modification can directly or indirectly regulate the stability and activation of PD-L1 mRNA. In Non-Small Cell Lung Carcinoma(NSCLC), METTL3-mediated m6A modification destabilizes PD-L1 mRNA, resulting in a reduction of PD-L1 expression (122). Furthermore, METTL3 can also regulate the ubiquitination of PD-L1 by controlling LINC02418 in NSCLC, thereby downregulating the expression of PD-L1 (85). Additionally, METTL3 mediates m6A modification of circIGF2BP3, upregulating the expression of PKP3, which enhances the stability of OTUB1 mRNA and increases the abundance of PD-L1 by reducing the ubiquitination of PD-L1 (123). Conversely, within the context of breast cancer, the m6A modification catalyzed by METTL3 not only bolsters the longevity of PD-L1 mRNA transcripts but also facilitates their transcriptional activation, a process that hinges on the recognition of the m6A mark by the IGF2BP3 protein (124). YTHDF3 reads and destroys m6A-modified CBX1 mRNA in nasopharyngeal carcinoma, inhibiting the upregulation of PD-L1 mediated by the IFN-γ/STAT1 signal (125). In HNSCC, TRMT61A-mediated tRNA-m1A modification upregulates the expression of PD-L1, which may be accomplished by regulating INFγ (126). m5C methylation reader YBX1 is related to PD-L1 expression levels in pancreatic ductal adenocarcinoma (50). In colorectal cancer, m6A-modified IFIT1 upregulates the expression of PD-L1 by reducing the ubiquitination and degradation of PD-L1 (127). Similarly, in cholangiocarcinoma, METTL14 mediates m6A modification of Siah2 and promotes the degradation of Siah2 upon reading by YTHDF2, inhibiting the ubiquitination of PD-L1 mediated by Siah2, ultimately leading to an increase in PD-L1 expression levels (128). It is worth mentioning that in colorectal cancer, the metabolite S-adenosylmethionine of methionine promotes the occurrence of m6A modification in cancer cells and enhances the translation of PD-L1 (129). m6A-regulated lncRNA has been proven to be associated with PD-L1 expression in various tumors (130–133). In pancreatic cancer, METTL3 increases the expression of PD-L1 by upregulating the expression of lncRNA MALAT1 in cancer cells (134). In hepatocellular carcinoma(HCC), intestinal bacterial lipopolysaccharide regulates the expression of PD-L1 on the surface of cancer cells by upregulating METTL14 and METTL14-mediated m6A modification of MIR155HG, modulating the miR-223/STAT1 axis (135). We present a summary of the effects of m6A modifications on PD-1/PD-L1 that have not been previously mentioned in this review (**Table 3**).

**Table 3:** Regulation of PD-L1 by m6A modification.

Regulators	Cancer type	Function	References
METTL16	colorectal cancer	reduce PD-L1 expression	(136)
	pancreatic ductal adenocarcinoma	reduce PD-L1 expression	(89)
METTL3	ccRCC	increase PD-L1 expression	(137)
IGF2BP1	colon cancer	increase PD-L1 expression	(96)
	HCC	increase PD-L1 expression	(18)
IGF2BP3	bladder cancer	increase PD-L1 expression	(138)
YTHDF1	NSCLC	reduce PD-L1 expression	(139)
YTHDF2	NSCLC	reduce PD-L1 expression	(139)
	lower-grade glioma	increase PD-1 expression	(140)
ALKBH5	gioblastoma mltiforme	increase PD-L1 expression	(105)
	intrahepatic cholangiocarcinoma	promote the expression of PD-L1 on monocytes/macrophages	(141)
	HCC	recruit PD-L1+ macrophages	(103)
FTO	OSCC	increase PD-L1 expression	(142)

### CTLA-4

CTLA-4, like PD-1, is a non-redundant checkpoint that inhibits the proliferation and activation of T cells. Therapy targeting the CTLA-4 pathway has been implemented in the field of immunotherapy and may be synergistically combined with PD-1 inhibitory therapy for specific cancer types (111). There are no definitive research results regarding how RNA modifications regulate CTLA-4, but we have found that RNA modifications are associated with the expression levels of CTLA-4 and can predict the outcomes of CTLA-4 blockade immunotherapy. The m6A-modified reader YTHDF2 has been proven to be positively correlated with the expression of CTLA-4 in low-grade glioma (140). In the high-risk group related to m5C-lncRNA, CTLA-4 is highly expressed (143). In tumors such as pancreatic adenocarcinoma, hepatocellular carcinoma, and pancreatic cancer, the scores of m6A-modified regulatory genes are significantly correlated with the expression of CTLA-4 and can be used to predict prognosis and response to immunotherapy (144–146). A study of m5C in diffuse large B-cell lymphoma has identified a correlation between m5C-affected genes and the modulation of immune checkpoint genes, specifically CTLA-4 and PD-L1. In prostate cancer, CTLA-4 is also found to be differentially expressed in two m5C immune subtypes and is related to the degree of immune cell infiltration (76). Similarly, in the immunological model calculated based on the regulatory genes of m7G such as CDK1, ANO1, and PDGFRA, CTLA-4 is highly expressed in the high-risk group (147). Another similar study shows that m7G is not only related to immune checkpoints such as CTLA-4 but also can predict the effects of immunotherapy (148). In addition, a low score based on the methylation enzyme scoring established by m6A/m5C/m1A/m7G regulatory genes is related to the positive expression of CTLA4 (149).

In this section, we have summarized the regulatory effects of RNA modifications on the immune checkpoint PD-L1, as well as the correlation with CTLA-4 expression and its predictive role in therapeutic efficacy. It provides new insights into the understanding of tumor immune evasion mechanisms. The research indicates that RNA modifications influence the expression of PD-L1 and CTLA-4 through various mechanisms, including the regulation of mRNA stability, transcriptional activation, and interactions with specific proteins. These findings offer new perspectives for anti-PD-1/PD-L1 therapy and reveal the research value of RNA modifications in anti-CTLA-4 therapy.

## The regulatory role of RNA modification in ICI therapy

**Figure 3:**
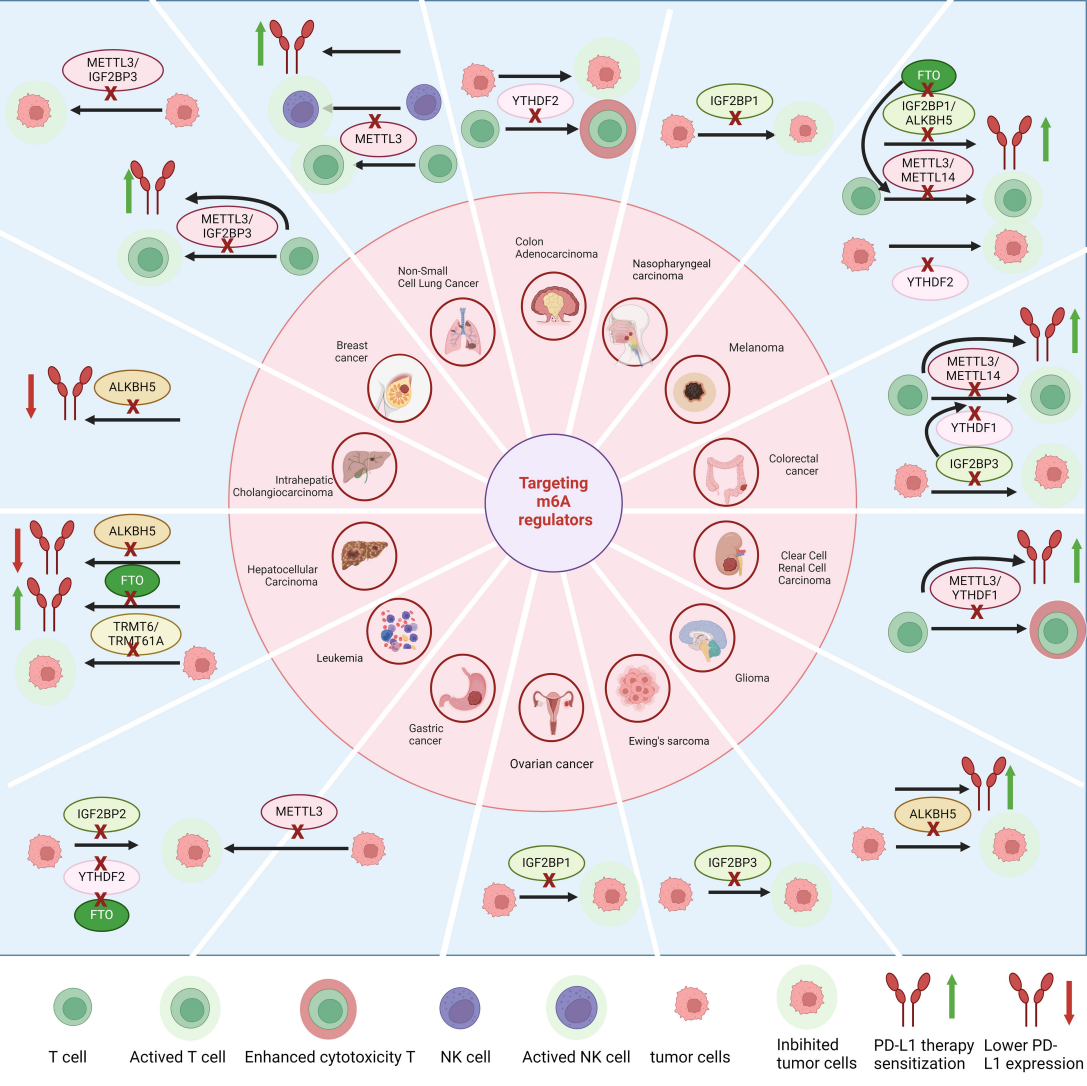
Targeting regulators with m6A modifications can enhance T cell infiltration and tumor-killing effects in various types of cancer. It also regulates the expression of PD-L1 and the therapeutic effects of anti-PD-L1 treatment, while inhibiting tumor growth.

RNA modifications exhibit differential impacts on PD-1 blockade therapy across various tumors. Targeting specific RNA modification regulators can enhance the efficacy of PD-1 blockade therapy (**Figure 3**). In NSCLC, METTL3-driven m6A methylation leads to the destabilization of PD-L1 mRNA, which reduces the therapeutic efficacy of anti-PD-1/PD-L1 interventions; however, the knockout of METTL3 increases immune cell infiltration and enhances the therapeutic efficacy of anti-PD-1/PD-L1 (122). Similarly, the deletion of METTL3 or METTL14 in immune-resistant melanoma tumor cells makes the tumor sensitive to immunotherapy (79). Targeting METTL3 in NAFLD-HCC and NSCLC can improve the effectiveness of PD-1 therapy. In the NAFLD-HCC mouse model, the knockout of METTL3 in conjunction with anti-PD-1 therapy synergistically suppressed tumor growth, resulting in a reduction of over 90% in both tumor volume and weight (83). In NSCLC, the suppression of METTL3 can enhance the sensitivity of tumor-bearing mice to anti-PD-1 treatment, and patients with NSCLC exhibiting low METTL3 expression have a more favorable prognosis with immunotherapy (122). In thyroid cancer, high expression of METTL3 in tumor cells inhibits the demethylation of CD70mRNA, maintains the degradation of transcripts mediated by YTHDF2, thereby releasing T cells from suppression and enhancing the efficacy of PD-1 blockade (86). Similar observations have been made in melanoma and lung cancer, where high expression of METTL3 in macrophages is beneficial for immunotherapy (63). Targeting the YY1-CDK9 transcription elongation complex in glioblastoma results in lowered METTL3 expression, which in turn, enhances the therapeutic impact of PD-1 blockade (150). Abrine treatment inhibits IFN-γ-induced m6A modification, thereby regulating JAK1/STAT1 and suppressing the expression of PD-L1. Combined therapy with PD-1 blockade can inhibit tumor growth (151). Targeting YTHDF1 in colorectal cancer can relieve the inhibition of CD8^+^ T cells and enhance the efficacy of anti-PD-1. Targeting YTHDF1 significantly reduced the resistance to anti-PD-1 therapy in the MC38 tumor model, leading to better prognosis for tumor-bearing mice. Similar observations were made in mice with the CT26 tumor model, which is insensitive to PD-1 therapy; the deletion of YTHDF1 in CT26 cells followed by anti-PD-1 treatment markedly inhibited tumor growth (91). Knocking out ALKBH5 in glioma or targeting it with IOX1 reduces the expression of the PD-L1 protein, inhibits tumor growth, extends the survival of mice, and enhances these effects of PD-1 blockade therapy (105). Similarly, inhibiting ALKBH5 in melanoma enhances the efficacy of PD-1 blockade, patients with low expression of ALKBH5 are more likely to benefit from PD-1 blockade therapy (31). The use of FTO inhibitors in HCC and melanoma can enhance immune activation and sensitivity to anti-PD-1 treatment (99, 152). Targeting circular RNA circRHBDD1 can block its m6A-dependent mediated rapid translation of PIK3R1 and improve the efficacy of anti-PD-1 therapy (153). NSUN2-mediated m5C methylation modulates TREX2 expression, thereby suppressing the cGAS/STING pathway and contributing to resistance against PD-1 checkpoint blockade (51).

In this part, we have explored the regulatory role of RNA modifications on PD-1 blockade therapy across different types of tumors. By affecting the expression of genes involved in immune activation and suppression, RNA modifications have significantly impacted the therapeutic efficacy of immune checkpoint blockade and have provided ideas for specific treatment plans: by targeting specific RNA modification regulators, we may be able to increase the response rate to immunotherapy and overcome patient resistance to existing treatments.

## RNA modification and prediction of immunotherapy efficacy

Genes related to the RNA modifications m6A, m1A, m5C, and m7G can predict tumor sensitivity to immunotherapy, either individually or in combination. By conducting bioinformatics analysis on many samples in databases, key genes are screened, and corresponding scores are established. For instance, in HCC, an m6A score derived from m6A-associated feature genes categorizes patients into distinct risk groups. The high-risk cohort demonstrates increased immune evasion and immune system dysregulation, correlating with heightened responsiveness to anti-CTLA-4 and anti-PD-1 therapies (154). The m6A score has been studied in multiple tumors but varies depending on the analysis method and selected genes. Patients with oral squamous cell carcinoma exhibiting a high m6A score are more likely to experience enhanced efficacy from treatments targeting the PD-1 and CTLA-4 pathways (155), lung squamous cell carcinoma (156), soft tissue sarcoma (157), gastric cancer (102), and hepatocellular carcinoma (158), while a low m6A score suggests greater sensitivity to immunotherapy in thyroid cancer (159), NSCLC (160), follicular lymphoma (161), breast cancer (162), and head and neck squamous cell carcinoma (163). Additionally, the score of m6A methyltransferase regulators can effectively predict the efficacy of immunotherapy in urothelial cancer patients (164).

A prognostic model established using m6A-related lncRNA suggests that esophageal cancer in the low-risk group responds better to immunotherapy (165). Similarly, a model established using m5C-related genes can also evaluate prognosis and immune therapy efficacy, with liver and pancreatic cancers with lower m5C scores being more sensitive to anti-CTLA-4 therapy, and pancreatic cancer also being sensitive to anti-PD-1 therapy (166). A score based on m7G indicates that colorectal cancer and rectal cancer in the low-scoring group are more sensitive to anti-PD-1 therapy (119, 121). Lung adenocarcinoma with a low score has a higher immune prognostic score (120). Interestingly, in low-grade glioma, the high m7G score group is sensitive to anti-PD-1 treatment, while the low m7G score group is more sensitive to anti-PD-L1 (167). In colorectal cancer, a low m1A score suggests a better prognosis with anti-PD-L1 treatment (77). Lung adenocarcinoma with a low m1A score has a lower TIDE score, indicating greater sensitivity to immunotherapy (78).

In addition to establishing models based on the scores of single RNA modifications, analyzing multiple RNA modification genes simultaneously can also establish effective predictive models. In cervical cancer, a prognostic model established using m6A/m5C/m1A indicates that the high-risk group is more sensitive to anti-CTLA-4 treatment (168). In colon cancer, the low-risk group is more sensitive to anti-CTLA-4 and anti-PD-1 treatments (169). In HCC, a methylation score composed of m1A/m5C/m3C/m6A suggests that a low score is sensitive to anti-PD-L1 therapy (170). The Writer-Score established according to m1A and m6A RNA modification enzymes indicates that a low score is associated with better outcomes in immunotherapy (171).

Through bioinformatics analysis of large-sample databases, we have been able to screen for key genes and establish corresponding scoring systems. These scoring systems have demonstrated the potential to predict responses to immunotherapy across various types of tumors, offering new tools for personalized medicine. However, many scoring systems still require further validation and research to explore their applicability in oncology and immunotherapy strategies.

## RNA modification regulators as therapeutic targets

Therapeutic interventions targeting RNA modification-related genes or proteins have been extensively studied, and numerous effective drugs have been developed. For instance, metformin can specifically inhibit FTO and block its demethylation effect on m6A modification (172). Targeting specific regulatory genes or proteins of RNA modification can enhance the therapeutic efficacy of immunotherapy in certain types of cancer. For example, targeting ALKBH5 in melanoma can enhance the effectiveness of PD-1 blockade (31). Lower levels of METTL3 in NSCLC are also associated with better outcomes from anti-PD-1 therapy (122). Moreover, targeting specific RNA modification regulators’ genes or proteins can directly exert tumor-suppressive effects. For example, miR-4429, which targets METTL3 in gastric cancer, can inhibit the proliferation of GC cells and induce apoptosis (14). Although the vast majority of drug treatments target m6A regulatory genes or proteins, we found that targeting the m1A methyltransferase complex TRMT6/TRMT61A in HCC with Hiram can effectively inhibit the progression of HCC (32), indicating that targeting other RNA modification regulatory genes or proteins also has therapeutic significance. We have summarized the specific targeted drugs and their mechanisms of action (**Table 1**).

This section of our study extensively explores therapeutic interventions targeting genes or proteins associated with RNA modifications and outlines the development of a series of effective drugs. Targeting regulators such as METTL3, METTL14, IGF2BP3, or YTHDF1 can alleviate T-cell suppression in melanoma, colorectal cancer, non-small cell lung cancer (NSCLC), and breast cancer. Targeting regulators like METTL3, METTL14, the IGF2BP family, ALKBH5, or FTO can enhance the efficacy of PD-1 blockade therapy in renal clear cell carcinoma, colorectal cancer, melanoma, NSCLC, breast cancer, hepatocellular carcinoma, and intrahepatic cholangiocarcinoma. Inhibition of tumor cell proliferation can be achieved by blocking METTL3, TRMT6/TRMT61A, the IGF2BP family, YTHDF2, or ALKBH5. Targeting “writers” such as METTL3 or METTL14 blocks the formation of m6A and thus can play a role in multiple tumor-suppressing or immune therapy-enhancing effects; however, this may also greatly impact normal physiological functions and lead to severe side effects. RNA modifications have a considerable number of “readers,” so targeting specific “readers” in specific tumors may achieve precise therapeutic effects with lower side effects, but it may need to be specific to certain tumors and may not be universally applicable. Targeting “erasers,” mainly FTO and ALKBH5, seems to primarily enhance PD-1 blockade therapy and inhibit tumor cells, being key to combined immunotherapy. However, targeting FTO and ALKBH5 results in the inability to remove m6A, which may also lead to severe side effects.

## Discussion

In this review, we primarily discuss the relationship between m6A, m1A, m5C, and m7G modifications and tumor immunity and immunotherapy, and summarized the regulation of immune cells and immune checkpoints, as well as drug treatment targeting RNA modification regulators and immune prediction based on the four RNA modifications. Research related to RNA modifications has the following limitations: m6A is the most abundant modification in mRNA and has been extensively studied; however, the physiological processes in which m6A is involved are not fully understood, and there may be undiscovered regulatory proteins related to m6A modification. The specific roles of m1A, m5C, and m7G in tumors have been insufficiently studied, possibly due to variations in modification sites and abundance. There are also limitations and challenges in tumor therapy related to RNA modifications: some proteins among RNA modification-related proteins have dual identities, and the same regulatory factor may have multiple roles, possessing both oncogenic and tumor-suppressive effects, requiring specific research to provide solutions. For example, knocking out METTL3 in melanoma can inhibit tumor development and increase the infiltration of CD8^+^ T cells (79), but another study has shown that the absence of METTL3 expression in macrophages can promote the growth and metastasis of melanoma and weaken the efficacy of PD-1 blockade (63). Additionally, RNA modifications are widely involved in physiological activities, and it is challenging to target RNA modifications in the tumor microenvironment without affecting normal physiological processes. Researchers have obtained drugs with high specificity through complex drug design and optimization, but this issue has not been resolved. Moreover, although many RNA modification regulators have been identified, only a few can be used as therapeutic targets for cancer treatment. Finally, current research is mainly focused on mouse models, and there may be differences in the physiological activities and tumor therapy based on mRNA between mice and humans, requiring more research to clarify the specific situation.

Despite certain achievements of drugs targeting RNA modifications in preclinical models, no related drugs have yet entered the clinical research phase. Future research in the following areas may be helpful: designing drugs to specifically target RNA modification regulators, especially in the tumor microenvironment, to reduce the impact on normal tissues; exploring appropriate drug dosages to balance efficacy and side effects; continuing drug safety research to assess the side effects of different targeted drugs; developing new therapeutic targets or drugs to improve treatment safety; and exploring the combined application of RNA modification-targeting drugs with other drugs in tumor therapy. In addition, tumor vaccines are also related to RNA modifications such as m6A and have therapeutic potential. Introducing encoded mRNA molecules into the bodies of tumor patients, translating them into proteins with anti-tumor effects in the body to trigger anti-tumor immune responses is a new treatment method (173). RNA modifications play a key role in cancer vaccines. Through RNA modifications, the immunogenicity of RNA vaccines can be eliminated, and the rapid translation of anti-tumor proteins may be promoted, improving anti-tumor effects. This direction has considerable potential for development. Furthermore, lifting the immune suppression caused by RNA modifications may help improve the efficacy of adoptive immunotherapy. By lifting the toxicity suppression and infiltration suppression of T and NK cells caused by RNA modifications, the corresponding tumor-killing ability can be restored, which can be used for ex vivo expansion and then re-introduced into the patient’s body to play an anti-tumor role. Lifting the immune suppression of the tumor microenvironment is beneficial for adoptive immune cells to clear the tumor. This could greatly enhance adoptive immunotherapy and holds value for research.
